# Bacterial Toxin–Antitoxin Systems: More Than Selfish Entities?

**DOI:** 10.1371/journal.pgen.1000437

**Published:** 2009-03-27

**Authors:** Laurence Van Melderen, Manuel Saavedra De Bast

**Affiliations:** Laboratoire de Génétique et Physiologie Bactérienne, IBMM, Faculté des Sciences, Université Libre de Bruxelles, Gosselies, Belgium; Baylor College of Medicine, United States of America

## Abstract

Bacterial toxin–antitoxin (TA) systems are diverse and widespread in the prokaryotic kingdom. They are composed of closely linked genes encoding a stable toxin that can harm the host cell and its cognate labile antitoxin, which protects the host from the toxin's deleterious effect. TA systems are thought to invade bacterial genomes through horizontal gene transfer. Some TA systems might behave as selfish elements and favour their own maintenance at the expense of their host. As a consequence, they may contribute to the maintenance of plasmids or genomic islands, such as super-integrons, by post-segregational killing of the cell that loses these genes and so suffers the stable toxin's destructive effect. The function of the chromosomally encoded TA systems is less clear and still open to debate. This Review discusses current hypotheses regarding the biological roles of these evolutionarily successful small operons. We consider the various selective forces that could drive the maintenance of TA systems in bacterial genomes.

## Introduction

Although bacteria have long been known to exchange genetic information through horizontal gene transfer, the impact of this dynamic process on genome evolution was fully appreciated only recently using comparative genomics (reviewed in [1]). Bacterial chromosomes are composed of genes that have quite different evolutionary origins (reviewed in [2]). The set of genes that is preferentially transmitted vertically over long evolutionary time scales composes the core genome. Core genes are relatively well conserved among different monophyletic groups and encode the cellular core functions. These core genes are interspersed with groups of genes that have been acquired from other prokaryotic genomes by horizontal transmission. These genomic islands mostly originate from integration events of mobile genetic elements, such as insertion sequences, transposons, phages, and plasmids. They might, therefore, be found in phylogenetically distant species and are not conserved among different isolates belonging to the same bacterial species. This set of genes constitutes the flexible genome.

Both gene influx and efflux processes are important in shaping bacterial-genome content. A vast majority of horizontally transferred genes are quickly lost after integration [3], although some remain interspersed in the genome (reviewed in [2]). Bacterial toxin-antitoxin (TA) systems appear to be subjected to this flux. Indeed, these small gene systems are found in plasmids as well as in chromosomes, and they are thought to be part of the flexible genome [4]. Although their role, when they are located in plasmid, is fairly clear, the involvement in physiological processes of the TA systems' chromosomally encoded counterparts is still open to debate.

Here we discuss current hypotheses regarding the biological roles of chromosomally encoded TA systems and consider the various selective forces that could drive the maintenance of TA systems in bacterial genomes.

## Diversity and Abundance of Bacterial TA Systems

Bacterial TA systems are of two different types depending on the nature of the antitoxin; the toxin always being a protein. The antitoxin of type I systems is a small RNA (antisense or adjacent and divergent to the toxin gene) showing complementarity to the toxin mRNA (for recent reviews on type I systems, see [5],[6]). Type I antitoxins regulate toxin expression by inhibiting the toxin's translation. The toxins of type I systems are small, hydrophobic proteins that cause damage in bacterial cell membranes. In type II systems, the antitoxin is a small, unstable protein that sequesters the toxin through proteic complex formation (for a recent review on type II systems, see [7]). Much more information is available for type II systems, especially in terms of their biological roles. We will focus on the type II systems and use the term TA systems for brevity.

Type II TA systems are organised in operons, with the upstream gene usually encoding the antitoxin protein. The expression of the two genes is regulated at the level of transcription by the antitoxin–toxin complex. Nine families of toxins have been defined so far based on amino sequence homology [4]. Their targets and the cellular processes that are affected by their activities are shown in Table 1.

**Table 1:** The nine toxin families.

Toxin	Target	Activity	Cellular Process
CcdB	DNA gyrase	Generates DS breaks^1^	Replication
RelE	Translating ribosome	Induces mRNAs cleavage^2^	Translation
MazF	RNAs	Endoribonuclease	Translation
ParE	DNA gyrase^3^	Generates DS breaks^4^	Replication^5^
Doc	Translating ribosome	Induces mRNAs cleavage^6^	Translation^7^
VapC	RNAs	Endoribonuclease^8^	ND
ζ^9^	ND	Phosphotransferase^10^	ND^11^
HipA	EF-Tu^12^	Protein kinase^13^	Translation^14^
HigB^15^	Translating ribosome	mRNAs cleavage^16^	Translation^17^

The targets and the types of activities of the nine toxins as well as the cellular processes that are affected by the expression of the toxins are shown. This table is adapted from [7] except where indicated. ND, not determined.

1 The CcdB toxin does not generate double-strand breaks by itself. Overexpression of CcdB inhibits the re-ligation step of the DNA gyrase, a type II topoisomerase, which leads to the generation of double-strand breaks.

2 Overexpression of RelE induces cleavage of mRNAs at the ribosome A-site.

3,4 ParE was shown to poison DNA gyrase and to generate double-strand breaks in vitro.

5 As CcdB, it induces inhibition of cell division and therefore, it is assumed that it inhibits replication.

6 Overproduction of the Doc toxin activates the *relBE* TA system and indirectly causes mRNA cleavage [53].

7 Doc inhibits translation elongation by association with the 30S ribosomal subunit [54].

8 See [55]. Although VapC shows an endoribonucleolytic activity, it has not been reported whether or not VapC is able to inhibit translation.

9 The ζ toxin is part of a three-component TA system (ω−ε−ζ) in which the antitoxin and autoregulation properties are encoded by separate polypeptides.

10 See [56].

11 At a high overexpression level, the ζ toxin inhibits replication, transcription, and translation, eventually leading to cell death [57]. However, the specific target(s) is (are) unknown.

12 See [34].

13 See [33].

14 See [32],[33],[34].

15 The genetic organisation of the *higBA* system is unusual; the toxin gene is upstream of the antitoxin gene in the operon.

16,17 See [40],[58].

Comprehensive genome analyses have highlighted the diversity in the distribution of TA systems [4],[8],[9]. Some genomes such as that of *Nitrosomonas europeae*, *Sinorhizobium meliloti*, and *Mycobacterium bovis* contain more than 50 putative TA systems. Some others contain no or very few (less than three) putative TA systems, such as *Rickettsia prowazeki*, *Campylobacter jejuni*, or *Bacillus subtilis*. No correlation between the number of TA systems, the lifestyle, the membership of a phylum, or the growth rate (as it was proposed [4]) could be drawn [9]. Another level of diversity in distribution of TA systems among bacteria is added when comparing the occurrence of TA systems between different isolates of the same species. Table 2 shows the distribution of the nine toxins in seven sequenced *Escherichia coli* strains.

**Table 2:** Occurrence of toxin homologues in seven *E. coli* chromosomes.

Toxin	*E. coli* strains						
	O157:H7 Sakaï	O157:H7 EDL933	536	CFT073	UTI89	MG1655 K-12	B7A
CcdB	+	+	+	+	−	−	+
RelE	−	−	−	−	−	+	−
MazF	+(2)	+(2)	+	−	+	+(2)	−
ParE	−	+	−	−	−	−	−
Doc	−	−	−	−	−	−	−
ζ	−	−	−	−	−	−	+
HipA	−	+	+	+	−	+	+
HigB	−	−	+(2)	+	+(2)	−	−
VapC	−	−	−	−	−	−	−

Homologues of the nine toxins were identified by Psi-Blast [59] in the chromosomes of seven *E. coli* isolates. Homologues are either present in one copy (+), in two copies (+(2)) or absent (−).

As an example, homologues of the CcdB, MazF, and HipA toxins are frequently represented (at least in five chromosomes), whereas others appear to be absent (Doc and VapC) or present in only one chromosome (RelE and ParE). This implies that these TA systems were integrated in chromosomes through horizontal transfer, most probably in very recent events. The copy number of TA systems within one genome may also vary from one isolate to another. For instance, the MazE and HigB toxins are present in two copies in at least two genomes. Thus, TA systems are part of the flexible genome. They might be located in cryptic prophages such as *relBE* in the *E. coli* K-12 Qin prophage or constitute genomics islets by themselves such as *ccd_O157_* [10].

## TA Systems: Just Selfish Entities?

Table 2 implies that the integrations of TA systems in *E. coli* chromosomes are recent events, because the distribution of the different TA systems varies from one isolate to another, raising the possibility that chromosomally encoded TA systems might have no physiological function. An attractive possibility is that TA systems act as selfish entities. Toxin and antitoxin genes show a strong interdependence (the functionality of the antitoxin is indispensable for the survival of cells carrying the toxin gene). They are closely linked, and they are capable of moving from one genome to another through horizontal gene transfer, as well as maintaining themselves in bacterial populations even at the expense of their host cell, at least when they are encoded in plasmids. Indeed, their stabilisation properties might be a consequence of their selfish behaviour (see below).

## TA Systems: More than Selfish Entities?

### Plasmid-Encoded TA Systems and Plasmid Fitness

**Figure 1:**
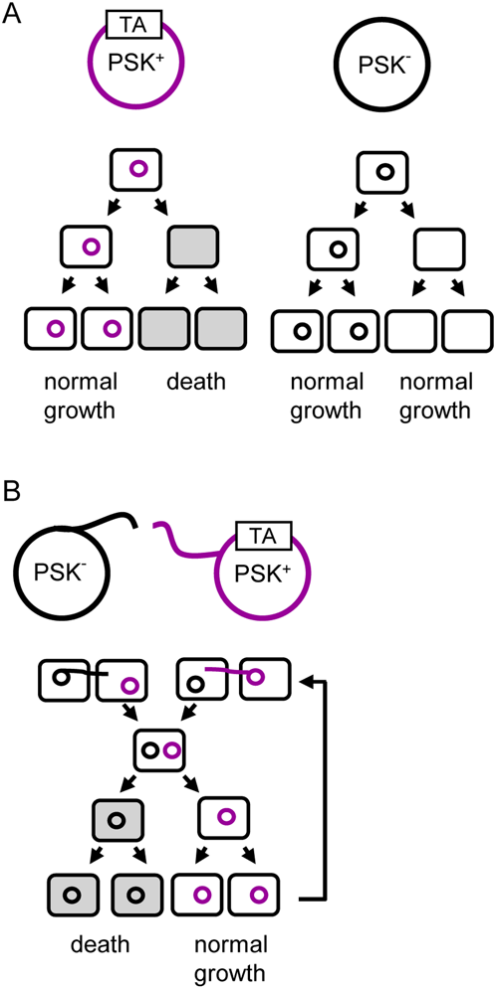
Advantage conferred by plasmid-encoded TA systems. (A) Vertical transmission. TA systems increase plasmid prevalence in growing bacterial populations by post-segregational killing (PSK). PSK^+^ plasmid is shown in purple, left panel. Daughter bacteria that inherit a plasmid copy at cell division grow normally. If daughter bacteria do not inherit a plasmid copy, degradation of the labile antitoxin proteins by the host ATP-dependent proteases will liberate the stable toxin. This will lead to the selective killing of the plasmid-free bacteria (in gray). When considering only vertical transmission, TA systems increase the prevalence of the plasmid in the population as compared with plasmids devoid of TA systems (PSK^−^ plasmid in black, right panel). (B) Horizontal transmission. Plasmid–plasmid competition. The PSK^+^ plasmid (in purple) and the PSK^−^ plasmid (in black) belong to the same incompatibility group and are conjugative. Under conditions in which conjugation occurs, conjugants containing both plasmids are generated. Because the two plasmids are incompatible, they can not be maintained in the same bacteria. The “loss” of the PSK^+^ plasmid will lead to the killing of bacteria containing the PSK^−^ plasmid through the PSK mechanism (in gray), thereby outcompeting the PSK^−^ plasmid. On the contrary, the loss of the PSK^−^ plasmid will be without any deleterious effect on the PSK^+^ plasmid. Through multiple events of conjugation, the fitness of the PSK^+^ plasmid will be increased (arrow).

Natural plasmids are often present in bacteria at very low copy number (one copy per chromosome). They are also able to spread by conjugation or mobilization with the help of other conjugative plasmids. They thus constitute a substantial proportion of the flexible genome and contribute importantly to bacterial evolution. TA systems increase the plasmid prevalence (number of plasmid-containing cells/total number of cells) in growing bacterial populations by selectively eliminating daughter cells that did not inherit a plasmid copy at cell division [11],[12] (Figure 1A). This post-segregational killing mechanism relies on the differential stability of the toxin and antitoxin [13],[14]. In daughter bacteria devoid of a plasmid copy, because TA proteins are not replenished, the antitoxin pool rapidly decreases, freeing the stable toxin. These plasmid-free bacteria will eventually be killed by the deleterious activity of the toxin. Plasmid-encoded TA systems are also called addiction modules [15], since this property renders the cell addicted to antitoxin production and therefore to the TA genes. Cooper and Heinemann [16] showed that TA systems might also function in plasmid–plasmid competition, as proposed for restriction-modification systems in the “selfish theory” of Kobayashi and colleagues [17],[18]. They showed that plasmid-encoded TA systems allow a conjugative plasmid (PSK^+^ plasmid) to outcompete a conjugative plasmid belonging to the same incompatibility group (identical replicon) but devoid of the TA system (PSK^−^ plasmid) [16]. Therefore, TA systems increase the relative fitness of their host DNA molecules by eliminating competitor plasmids in the bacterial progeny through post-segregational killing (Figure 1B). Mathematical models demonstrate that the post-segregational killing phenomenon allows the propagation of TA systems in bacterial populations, independently of their original frequencies [19]. This might provide rational explanation for the evolutionary success of TA systems.

### Chromosomally Encoded TA Systems

Some chromosomally encoded TA systems might be integrated in host regulatory networks and thereby confer a fitness advantage to the bacterial-host cells and/or populations. Several models supporting this view have been proposed.

#### A secure way to survive: Being integrated in host regulatory networks?

The **programmed-cell death model** is based on the study of the chromosomally encoded *mazEF* TA system of *E. coli* (reviewed in [20]). *mazEF*-mediated programmed cell death was observed by Engelberg-Kulka and colleagues under a wide variety of unrelated stressful conditions (e.g., amino-acid starvation, short-term antibiotic treatments, high temperature, and oxidative shock). Stress conditions are thought to affect the production of the *mazEF*-encoded proteins in a manner dependent on ppGpp, an alarmone synthesised under starvation [21]–[24] and through a quorum-sensing-like small peptide (extra-cellular death factor or EDF) [25]. This particular combination of stress conditions and EDF is thought to shut off *mazEF* transcription and lead to MazF toxin liberation as a consequence of MazE degradation by the ClpAP ATP-dependent protease. The outcome of this activation has been shown to be fatal for at least 95% of the bacterial population. Altruistic death of a fraction of the bacterial population is proposed to provide nutriments for the siblings. The molecular mechanisms underlying this proposed stochastic activation, as well as those by which killing is achieved, are still unknown. Whether MazF induces cell lysis also remains to be established.

The **growth-modulation model** is built on data mostly obtained on the *E. coli relBE* system and to a lesser extent on *mazEF* and *chpB* (which encodes a toxin homologous to MazF) [26],[27]. This model relies on the primary observation that amino-acid starvation inhibits cell growth without leading to cell death [26], in contrast with the programmed cell-death model. However, growth inhibition was subsequently shown to be independent of the presence of *relBE*, *mazEF*, *chpB*, and two other type II systems [28]. Nevertheless, upon amino-acid starvation, the rate of translation drastically drops in a wild-type *E. coli* strain and to a lesser extent in a Δ*relBE* mutant strain [26]. Gerdes and collaborators therefore proposed that *relBE* is a stress-response module that functions in quality control of gene expression to regulate the global level of translation, together with the *trans*-translation *ssrA* system [29]. Amino acid starvation activates *relBE* transcription through the Lon-dependent degradation of RelB and in a ppGpp-independent manner. As a consequence, RelE inhibits translation and induces a dormant state until favourable growth conditions return. Data obtained on *mazEF* and *chpB* by the group of Gerdes are consistent with the growth-regulator model and disagree with the programmed cell-death model [27], although each model could be true under different circumstances [21].

The **persistence model** describes an epigenetic trait that allows a small fraction of bacteria (∼10^−6^) to enter into a dormant state that renders them able to survive stress conditions, notably antibiotic treatments (reviewed in [30]). A nontoxic mutant of the HipA toxin (*hipA7*) has been shown to confer high persistence in *E. coli* [31]. Mutations abolishing the production of the ppGpp alarmone eliminated the high persistence phenotype, suggesting that *hipA7* might induce a high level of ppGpp [31]. Persistence and toxicity might be independent, because the HipA7 mutant seems to be less efficient for inhibition of macromolecule synthesis as compared to the wild-type HipA [32]. However, the protein kinase activity of HipA was shown to be required for persistence and growth arrest [33]. The central elongation factor Tu (EF-Tu) was recently shown to bind and to be phosphorylated by HipA [34]. EF-Tu in its nonphospohorylated form catalyses the binding of aminoacyl-tRNAs to the ribosome. Phosphorylation of EF-Tu by HipA might lead to translation inhibition [34] and therefore to ppGpp synthesis. Single-cell analysis revealed that several TA systems are up-regulated in persister cells [35]. The biological meaning of this observation remains unclear, since the deletion *mazEF* and *relBE* did not impair persister frequency under ofloxacin (a fluoroquinolone) or mitomycin C treatments. However, the Δ*hipBA* mutant strain was strongly affected (10- to 100-fold), showing that this TA system is involved in persistence [36]. The molecular mechanisms underlying this stochastic phenomenon are unknown.

The **development model** was proposed recently for fruiting body formation in *Myxococcus xanthus*. A homologue of the *mazF* toxin gene (*mazF-mx*), which is devoid of any *mazE* antitoxin gene homologue, was identified in the chromosome of *M. xanthus* [37]. The solitary *mazF-mx* toxin gene constitutes an interesting example of integration in host regulatory networks. *M. xanthus* forms multicellular structures called fruiting bodies under nutrient-starvation conditions. During this process, 80% of the population engaged in fruiting-body formation die by lysis; only 20% will develop into myxospores. The *mazF-mx* gene is integrated in a regulatory cascade controlled by the key developmental regulator MrpC, which presents a dual activity towards *mazF-mx*: it positively regulates *mazF-mx* expression at the transcriptional level and it negatively controls its endoribonuclease activity at the post-translational level by acting as its antitoxin. During vegetative growth, MrpC transcriptional activity is controlled negatively by its phosphorylation through a Ser/Thr protein kinase. When *M. xanthus* engages in fruiting body formation, MrpC transcription activity is activated most likely by a LonD-dependent cleavage. MazF-mx is then produced and cleaves mRNAs, thereby inducing cell death. *mazF-mx* is essential for fruiting body formation, because a Δ*mazF-mx* mutant shows a dramatic reduction of myxospore formation.

In the above models, chromosomally encoded TA systems are thought to be integral parts of their host genetic networks. *mazEF* has been extensively reported as being responsible for programmed cell death, although this observation failed to be reproduced in various labs and is still a subject of debate [26]–[28]. Nevertheless, TA systems are thought to allow cells and/or populations to cope with stress conditions, and should therefore confer a clear selective advantage in these conditions. Indeed, *mazF-mx* and *hipBA* appear to be essential components of host regulatory networks, since their deletion caused a drastic phenotype [36],[37]. However, it is less clear for *mazEF*, *relBE*, and *chpB* of *E. coli*, since no fitness gain could be attributed to their presence neither under stress conditions nor during post-stress recovery phases [28].

The two following models provide an alternative to the previous ones by illustrating how TA systems can confer selective advantages to their bacterial host without being integrated into regulatory networks.

#### TA systems in dynamic genome evolution

The **stabilisation model** proposes that because of their addictive characteristics, chromosomally encoded TA systems could act against large-scale deletion of otherwise dispensable genomic regions [38]. Super-integrons are plastic platforms composed of numerous gene cassettes (more than a hundred in the *Vibrio cholerae* super-integron) and repeat sequences (reviewed in [39]). Super-integrons encode many functions (e.g., antibiotic resistance). Super-integrons may advantage bacterial populations over long time scales by maintaining nonessential genes and allowing bacterial lineages to better cope with unpredictable changes of environmental conditions. Gene cassettes are excised, integrated, and rearranged by the action of the SI-encoded integrase. They contain in general a single gene devoid of promoter, except for the TA systems encoding cassettes. In this case, the entire TA operon is present in the cassette and is most likely expressed. Several TA systems from super-integrons belonging to various Vibrionaceae are able to stabilise otherwise unstable plasmids or large genomic regions in *E. coli* [38],[40],[41]. Moreover, super-integrons are extremely stable. Attempts to delete the super-integron of *V. cholerae* have failed, strongly suggesting that TA systems serve to stabilise the super-integron platform and counteract gene efflux (D. Mazel, personal communication).

While it becomes clear that TA systems in such genetic structures or in cryptic prophages such as *relBE* of Qin [42] have retained their stabilisation properties, the generalisation to more “classical” chromosomally encoded TA systems should be taken with caution. Although only a few systems have been tested (*E. coli dinJ-yafQ* and *ccd_O157_* systems), they appear to be unable to prevent large-scale deletion or to stabilise an otherwise unstable plasmid [10],[38]. Wide surveys of stabilisation properties of TA systems from various locations (mobile genetic elements, core, genomic islands, remnants) will test whether a correlation between stabilisation function and localisation exists.

**Figure 2:**
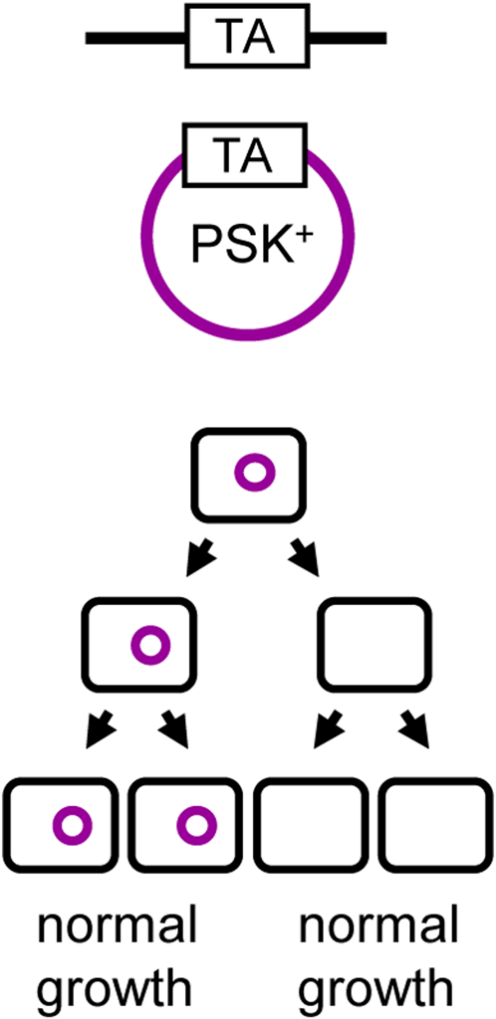
The anti-addiction model. The chromosomally encoded anti-addiction system is represented in black; the PSK^+^ plasmid in purple. In this model, the antitoxin of the chromosomally encoded TA system is able to counteract the toxin of the plasmid-encoded system. Therefore, daughter bacteria that do not inherit a plasmid copy at cell division will survive post-segregational killing.

The **anti-addiction model** proposes that chromosomally-encoded systems can selectively advantage their host in post-segregational killing conditions. In theory, chromosomally-encoded antitoxins sharing sufficient identity with homologous plasmid-encoded TA systems might act as anti-addiction modules by preventing post-segregational killing (Figure 2). The *ccd_Ech_* chromosomally encoded TA system of *Erwinia chrysanthemi* 3937 was shown to have this property with respect to its *E. coli* F plasmid–encoded *ccd_F_* homolog [43]. In an *E. coli* strain containing the *ccd_Ech_* system inserted in its chromosome (*ccd_Ech_* strain), no post-segregational killing was observed upon the loss of a plasmid carrying *ccd_F_*. Moreover, competition experiments showed that under post-segregational killing conditions, the *ccd_Ech_* strain had a selective advantage compared to the wild-type strain. Therefore, the fitness advantage conferred by the newly acquired anti-addiction module under post-segregational killing conditions might allow its fixation in the bacterial population. In turn, the plasmid-encoded system will lose its addictive character. On the one hand, variants able to evade anti-addiction modules are expected to be selected and out-compete their post-segregational killing–defective relatives. Anti-addiction might thus be one of the evolutionary forces driving selection of the plasmid encoded TA systems. On the other hand, chromosomally encoded TA systems might lose their anti-addictive properties [10] and decay [44].

## Conclusions

There is no doubt that bacterial TA systems are evolutionarily successful entities. Some bacterial genomes harbour several dozen of them [4],[9]. Even obligatorily intracellular species that undergo massive genome reduction contain TA systems [9],[45]. There is increasing evidence that these small entities move between genomes through horizontal gene transfer. Their phylogeny is not congruent with the bacterial one [4],[46], and their distribution varies greatly between isolates belonging to the same bacterial species ([44],[46], Table 2), implying that TA systems are highly mobile. Pandey and Gerdes also reported recently that TA systems are preferentially associated with genomic islands [4]. However, how horizontally acquired TA systems are fixed within the population is not yet understood. One can argue that their addictive “selfish” characteristics enable them to be stabilised and refractory to gene efflux. As a consequence, in specific genomic locations such as plasmids or genomic islands, they may contribute to the maintenance of these structures in bacterial population by post-segregational killing and be subjected to selection. In other genomic locations, such as the core genome where they are not subjected to selection, some TA systems might accumulate mutations that reduce or inactivate their addictive properties simply by genetic drift. Indeed, deletion of both type II [24],[26],[47] and type I systems [48],[49] in *E. coli* K-12 was possible, at least under the conditions used in these experiments, suggesting that these systems have lost their addictive characteristics. Signs of “loss of addictive properties” were detected for several type I and II systems. For instance, the five copies of the type I *hok-sok* system located in the *E. coli* K-12 chromosome are inactivated by insertion sequences, point mutation, or large rearrangements [50], and the *ccd_O157_* system appears to undergo a degenerative process within the *E. coli* species [44]. Similar observations have been reported for restriction-modification systems that share the addiction and apparent mobility characteristics of TA systems [51],[52]. Another route for TA system evolution is their integration into host regulatory networks. This is exemplified by the MazF-mx toxin in *M. xanthus* that had been hijacked by the developmental network controlling fruiting-body formation. The canonical antitoxin has been replaced by a complex cascade of signal transduction proteins involving a Ser/Thr protein kinase and a transcriptional activator/antitoxin protein [37].

Many scenarios might occur depending notably on the bacterial species and the type of toxin. The TA field should avoid generalisation regarding the biological role of these interesting entities. These small modules are highly diverse and ubiquitous. They might have multiple biological roles, if any, that depend on their age, their genomic location, the nature of the toxin, and most likely on many not-yet-discovered factors that influence their evolution.
